# Misconceptions and beliefs around hormone replacement therapy after childhood hematopoietic stem cell transplantation: A qualitative study among women leukemia survivors

**DOI:** 10.1371/journal.pone.0283940

**Published:** 2023-04-11

**Authors:** Julia Vergier, Rachel Reynaud, Gerard Michel, Pascal Auquier, Blandine Courbiere

**Affiliations:** 1 Department of Paediatrics, Paediatric Endocrinology Unit, CHU Timone Enfants, Assistance Publique-Hôpitaux de Marseille (APHM), Marseille, France; 2 Aix-Marseille Univ, Institut National de la Santé et de la Recherche Médicale (INSERM), U1251, Marseille Medical Genetics (MMG), Marseille, France; 3 Department of Pediatric Hematology and Oncology, CHU Timone Enfants, Assistance Publique-Hôpitaux de Marseille (APHM), Marseille, France; 4 Aix-Marseille Univ, Research Unit Equipe d’Accueil 3279, Marseille, France; 5 Aix-Marseille Univ, EA 3279: CEReSS—Health Service Research and Quality of Life Center, Marseille, France; 6 Aix Marseille Univ, Avignon Univ, CNRS, IRD, IBME, Marseille, France; 7 Assistance-Publique des Hôpitaux de Marseille (APHM), Pôle Femmes-Parents-Enfants, Centre Clinico-Biologique AMP-CECOS, Plateforme Cancer et Fertilité ONCOPACA-Corse, Marseille, France; University of Palermo: Universita degli Studi di Palermo, ITALY

## Abstract

**Purpose:**

After childhood leukemia and hematopoietic stem cell transplantation, hormone replacement therapy is often required to induce puberty because of premature ovarian insufficiency. Observance of this kind of treatment in adolescents and young women seems quite poor, and literature about its acceptance remains scarce; in order to learn about their experience and to better understand their attitude towards hormone replacement therapy, we used qualitative methods.

**Design and patients:**

13 young women childhood cancer survivors completed an individual interview.

**Results:**

We report that the negative experience of leukemia may cause rejection of the treatment, closely related to infertility unacceptance. Misconceptions and lack of adequate information of hormonal treatment effects are also major barriers to a good compliance.

**Conclusions and implications for cancer survivors:**

Observance of hormone replacement therapy for young women childhood cancer survivors can be improved with a confidential patient-physician relationship, patient education, choice of galenic formulation according to personal preference, and psychological support during the long-time follow up.

## Introduction

Premature ovarian insufficiency (POI) is currently a main endocrine aftereffect of anti-neoplastic gonadotoxic treatment. Among women survivors of pediatric cancer overall, the estimated cumulative incidence of POI is 8–10% [1]. The main independent risk factor is hematopoietic stem cell transplantation (HSCT) in hematological malignancies [2]. Indeed, 44% to 100% of patients transplanted during childhood present iatrogenic POI [3]. Conditioning regimen (total body irradiation, Busulfan) and age/pubertal status at the time of HSCT play a major role on ovarian function, as do supposed ovary-targeted graft-versus-host disease [3]. Therefore, after childhood HSCT, hormonal replacement therapy (HRT) is often required to induce puberty, acquire secondary sexual characteristics, pubertal height spurt and peak bone mass. After puberty, adequate HRT is considered to improve sexual health/well-being in young women [4]. HRT long-term goals are to provide estrogen impregnation according to age, manage symptoms, improve quality of life and protect against adverse long-term hypoestrogenism-related outcomes. In terms of public health, HRT has now shown clear benefits effects on bone mineral density in the specific context of HSCT [5], but also protects from cardiovascular diseases in younger women since an independent association of early menopause with increased risk of cardiovascular disease events has been proven [6, 7]. No consensus has been reached yet on the potential impact of the drop in estrogen levels after menopause as a risk factor for cognitive decline and dementia [8]. Clinical efficacy seems related to early treatment [9]. When properly prescribed and followed up, the safety of HRT has been well demonstrated [10, 11] including for cancer survivors [12]. Practical guidelines have been published for puberty induction and subsequent follow-up [3, 13, 14]. Hormonal contraceptive such as daily combined pills may also be used as HRT in young women with iatrogenic POI, bringing the additional benefit of preventing pregnancy in case of resumption of ovarian activity.

Only very few studies focused on patients’ experience of HRT in case of hypergonadotropic hypogonadism after HSCT. Treatment compliance appears to be good in adults because of its simple administration and few adverse effects [15]. Observance of such treatment in adolescents and young women seems comparatively weaker in our experience, and little is found in the available literature about HRT after HSCT for childhood cancer survivors [16]. There is a need for greater knowledge of the patients’ experience and acceptance. The aim of this qualitative study was to explore young women’s point of view regarding HRT and to point out their knowledge by the mean of individual interviews.

## Materials and methods

### Participants

We recruited adult women (18–40 years-old) from the French prospective childhood cancer survivor LEA cohort [17]. The women included underwent HSCT for childhood or adolescent myeloblastic or lymphoblastic acute leukemia between 1990 and 2010, needed HRT for puberty induction or progression because of POI, and were monitored in the University Teaching Hospital of Marseille according to the schedule described in the LEA protocol [17]. POI was confirmed by both clinical and biological criteria, following the ESHRE guidelines [14]. Participants were excluded if their level of understanding was too poor to go through the semi-structured individual interview or if their written consent was not collected.

### Study design and analysis

From 2018 to 2020, we conducted a qualitative study split into three main phases. Stage one was the development of a personal interview guide after a systematic literature review, which has been partially modified after the first interviews according to participant feedbacks. The second stage involved a trained single-investigator (JV, presented to participants as a female medical doctor trained to qualitative studies and working in pediatric endocrinology) to run single semi-structured individual interviews using various open-ended questions to understand women’s point of view regarding HRT; the audio-recorded interviews were led face to face in hospital or by telephone by default. Phase three was data investigation of transcribed interviews using textual and discourse analysis by one data coder without software, making subdivisions of identified in advance themes and topics with key sentences, key words and participants quotations. Transcripts were not returned to participants unless they asked for it. Inclusion was stopped when data saturation was achieved and data collected from testimonials became repetitive; a minimum of 10 interviews was initially planned. This study conforms with the Enhancing the QUAlity and Transparency Of health Research (EQUATOR) network guidelines and the 32-item checklist criteria included in COREQ [18].

### Semi-structured interviews

Interviews involved themes and topics including women socio-demographic data, memory of the initial prescription as an adolescent and level of understanding at that time, ability to talk about treatment with healthcare professionals and relatives, quality of the relationship with the prescriber. A major part was then dedicated to knowledge and (mis)belief about HRT, past and present observance, obstacle and limitations to its daily compliance. Women were interviewed alone. The list of items can be found in more details in *S1 Appendix*.

### Ethical approval

This study is an ancillary study of the FERTILEA study [19], approved by the Regional Ethics Committee (Comité de Protection des Personnes Sud Méditerranée, 09/22/2017—N°IDRCB: 2017-A01596-47, registered at ClinicalTrial.gov/NCT 03583294). Written consents were obtained from each participant when individual interviews were conducted.

## Results

### Participants

Thirteen women were approached and completed the interview, none refused to participate (*Table 1*). Acute lymphoblastic leukemia was the most represented hematological malignancy (n = 7/13, 53.8%). HSCT occurred between 1993 and 2010 at a mean age of 7.4 (2.5–12) years. Most women were prepubescent at the time of HSCT (n = 10, 77%), except for 3, from 2 of whom had had menarche before introduction of the anti-neoplastic treatment (aged 12 at HSCT for both of them). Conditioning regimen mostly consisted in total body irradiation (12 Grays) and/or chemotherapy with high dose alkylating agents (Busulfan, Melphalan, Cyclophosphamide). None of the 13 women benefited from fertility preservation technique before HSCT.

**Table 1:** Description of participants.

Patient	PAST					PRESENT			
	Type of hematological malignancy	Age at SCT (*years*)	Conditioning regimen	Signs of puberty before SCT	Spontaneous menses after SCT	Age at investigation (*years*)	History of pregnancy	Children	Pregnancy desire
1	acute lymphoblastic leukemia	11	TBI + Chemotherapy	No	No	33	Oocyte donation	Yes	Yes
2	acute myeloblastic leukemia	2,5	TBI + Chemotherapy	No	No	26	0	No	Yes
3	acute lymphoblastic leukemia	7,5	TBI + Chemotherapy	No	Yes, erratic*	22	Spontaneous (miscarriage)	No	Yes
4	acute myeloblastic leukemia	12	TBI + Chemotherapy	YesS2	No	36	0	No	No
5	acute lymphoblastic leukemia	6	TBI + Chemotherapy	No	Yes, erratic*	32	0	No	Yes
6	acute myeloblastic leukemia	7	Chemotherapy	No	No	22	0	No	Yes
7	acute lymphoblastic leukemia	7	TBI + Chemotherapy	No	Yes, erratic*	23	Spontaneous (miscarriage)	No	Yes
8	acute myeloblastic leukemia	12	TBI + Chemotherapy	Yes	No	37	Oocyte donation	Yes	Yes
				R1					
9	acute myeloblastic leukemia	3	TBI + Chemotherapy	No	No	27	0	No	Yes
10	acute lymphoblastic leukemia	*NA*	TBI + Chemotherapy	No	No	32	0	No	Yes
11	Dendritic leukemia	12	Chemotherapy	Yes	No	21	0	No	*NA*
				R1					
12	acute lymphoblastic leukemia	4,5	TBI + Chemotherapy	No	Yes, erratic*	25	0	No	Yes
13	acute lymphoblastic leukemia	4,5	TBI + Chemotherapy	No	No	32	0	No	Yes

Abbreviations:

NA: *Not available*

TBI: Total body irradiation

S2: Breast bud forms; papilla and areola elevates to form small mound (Tanner’s classification)

R1: Menarche

*Erratic: irregular menses, mainly spaniomenorrhea

Participants mean age was 28.3 (21–37) years. For 10 women, AMH serum level was undetectable (*data not available for 3 women*) and antral follicular count ranged between 0 and 2 for all the women. Only one woman still presented erratic menses without taking any hormonal treatment at the time of interview, nine were taking HRT, while three were having the daily combined hormonal contraceptive pill (one wanted to have a “regular pill” to replace HRT, the two others didn’t want to get pregnant being young adults at the time and aware of possible resumption of ovarian activity). At the time of inclusion, 46.2% of women were single (n = 6). All but one stated pregnancy desire, in the short or long-term. Two women reported history of spontaneous pregnancies, with early miscarriage. Two others already had children conceived via assisted reproductive technology with oocyte donation. None of them used non-hormonal contraception associated with HRT.

Interviews lasted between 25 and 110 min (mean duration 70 min). Data saturation was achieved after 13 interviews.

### Treatment initiation for puberty induction or progression

Participants’ memory of HRT first medical prescription was poor. Most women vaguely remembered the first time HRT was discussed as a conversation between the healthcare provider and their parents. The feeling about this precise moment was ambivalent. Some women felt either left aside (“*No one told me directly*”, “*this seemed not about me*, *it was all for my parents*”), not personally concerned *(“I was young*, *I didn’t care*”), or not much upset (“*Compared to all these years of hospitalization*, *this was routine*”). However, some women chose not to remember: *“This is a part of my past life*, *I hardly manage to remember because it has been so difficult to live through this period*”. Overall, initial comprehension was quite poor: as adolescents, they believed “*HRT was recommended in order for them to have regular menses like other girls at school*”, but didn’t understand the direct link between anti-neoplastic treatment ovary outcomes and HRT.

### Current information and misinformation about HRT itself

Some attendees read the notice from the pill box to get information, very few looked for complementary data on the Internet. They all declared feeling free to ask the prescriber for any additional information, but very few had actually done so. There were mixed opinions about this, ranging from “*I’ve never myself asked any question about this treatment […] I didn’t need answers from the doctor*” to “*I’ve had neither adequate information nor the answers I was looking for*, *I’m disappointed”*.

HRT was almost only related to providing menstrual cycles: “*HRT is given to have real periods with fake hormones*”. Various HRT scheme were prescribed, combined, sequential, and alternation between both without any clear preference. None of the 13 women interviewed complained about dysmenorrhea. Libido variations were rarely associated with HRT. Information on HRT positive benefits (bone metabolism, cardiovascular function, urogenital trophicity or well-being) was lacking for almost every women: “*I really don’t know what this treatment stands for except for menses*”.

On the other hand, misinformation was considerable. One participant said “*The point of this treatment is to fall pregnant*”, when another thought exactly the opposite: “*It damages my ovaries*”. Three women spontaneously declared being “*afraid of gaining weight under hormonal replacement treatment*”.

### Communication about HRT with professionals and relatives

Although talking about cured leukemia and HRT with doctor was often described as easy (pediatricians, gynecologists, endocrinologists or pharmacist), only few women felt free to mention it to close relatives. Only two women from our cohort saw no inconvenience talking with almost everyone: “*This is not a taboo subject*, *it is my past*, *and it explains who I am today*”. Otherwise, this matter was generally avoided: “*I really don’t see the point*”; “*I would deeply hate it if my siblings knew about everything*, *people look at you differently when they know you have been sick*”. Discussion with their partner was frequently described as difficult by the young women, especially at the beginning of a relationship, underpinned by the fear of scaring him off.

### Observance and compliance

Daily medication was not described as a constraint by most women “*It is a routine*, *just like brushing my teeth*”, but sometimes also “*because I have to do so…*”. Regimen of administration was poorly discussed, but estrogens patches seemed generally not appreciated. Compliance to HRT was better when libido improvement was noticed and in case of intercourse trouble (dyspareunia, pruritus). Weak adherence has been mainly related to the regimen of administration: transdermal delivery system such as estrogens cream or patches were described as inconvenient (“*Sticky patches are uncomfortable*, *adhesive stays for days on the skin*”), whereas pills had a better tolerance and therefore acceptance (“*Tablets are much easier to take*, *it is just like any other woman on the pill*”).

For about half of the participants, HRT was felt as a direct repercussion of past leukemia: “*This treatment is a reminder of me being sick*”, “*It is a remainder of this disease that ruined my whole life*”. Observance was poorer if related to leukemia “*because this pill makes me different*, *I would like to be just like any other girl without this past*”, or to gonadotoxic neoplastic drugs “*I’m angry about my sterility*, *therefore it is too painful to accept this treatment*” leading to: “*I frequently forget taking the pill*, *as a conscious oversight*, *because deep-down I reject it*”.

Almost all women disrupted their own HRT administering routine at least once, without following any medical advice. The reasons for that were diverse: long-term oversight, side effects, or psychological rejection. One woman who stopped her HRT several times during adolescence and in adulthood confided about her attitude: « *When you have to have a chronic (daily*?*) treatment*, *there are many factors in your life that step in to impact your compliance*, *and you easily get bored with it*. *There should be meetings with doctors to remind key points*, *this revaluation is essential for HRT*, *we may be more attentive to news elements when we get older*”. After durable therapeutic disruption, some felt collateral effects (“*I was tired*, *I could not stay focused*, *I told myself something was wrong so I started again taking the pill and felt better*”), but then on the other hand some psychologically felt better without it: “*Quitting taking HRT took a lot of the burden off*”; “*I was a rebel*, *independent and free without it*”.

### The infertility issue

For every women, infertility was the hardest part to live with once leukemia was cured: *“That makes me different”*, *“This burden was*, *and currently still is too difficult for me to accept*” and also “*I’m haunted by the psychological baggage of sterility […] it gives me nightmares*”. One participant remembered vague words heard as a child during HSCT (“*Pregnancy won’t be possible”*), but most women only understood gonadal failure in late adolescence, mainly during repeated medical consultations. “*Everything happens when you are young*, *and there is no decent way to tell a teenage girl that she’s going to be infertile*”. Some participants (n = 2) understood their infertility at school, when reproduction linked to spontaneous menses was taught: “*Everything became clear after science class and this was a painful announcing*”. These both cases were associated with a dreadful personal experience followed by bad compliance to HRT.

## Discussion

Ovarian reserve damage after allogenic HSCT in prepubertal girls often leads to POI and infertility. The latter is part of the burden for childhood cancer survivors, especially for those who could not benefit from fertility preservation techniques. Our experience states that HRT compliance is far from being enough, and there are new lessons we can consider after these interviews to improve cancer survivors care.

### What we may deduce after this study…

#### Lack of adequate information is a major barrier to HRT adherence

Almost every participant reported not being familiar with the role of sex hormones on bone density, cardiovascular or neurocognitive health, and general wellbeing. For most women, HRT’s only outcome was to have regular menses, therefore taking the treatment was often considered as useless. HRT increases bone mineral density in such hypogonadal women especially after HSCT, and specific information should be repeatedly delivered according to patients’ maturity and level of understanding [5]. Besides, encountering unexpected side effects was also a barrier to daily medication.

#### Fighting against misinformation is a priority for better compliance

Mistaken beliefs were common and consistently lead to poor observance, in the same way as any other chronic treatment. “*Weight gain*”, “*damaging ovaries*”, are part of misconceptions that need to be sought and discussed during repeated medical appointments.

### Negative experience of the illness is linked to rejection of HRT

POI may be regarded as a grief. For women with major concern regarding fertility and who could not cope with iatrogenic ovarian failure after HSCT, HRT compliance was poor especially when the treatment highlighted the injustice of the situation “*Taking the pill makes me remember every day that I am infertile*”.

### What they seem to expect during their medical care…

#### As a child/adolescent: Specific explanation that differs from the one needed by the parents’

Some adolescents felt left aside in a “*grown-up talk*” while healthcare providers explained POI to theirs parents. Therefore, initial individual consultation without relatives should be considered, repeated later on during follow-up to match medical explanation to age-related questions and to the level of comprehension. Technical supports handed over in order to be taken home and read at a distance could help, for instance visual documents, illustrated booklets or cartoons for the youngest and leaflets for the oldest. For those who felt too young or who were not emotionally ready to receive this information chemotherapy, specific times must be later searched in an adolescent-physician face-to-face dedicated medical consultation. Besides, the establishment of pediatric-adult transition consultations may help in such difficult cases.

#### As an adult: Better psychological support

Each childhood cancer survivor facing POI after HSCT requires long-term psychological support after the end of chemotherapy, especially to overcome anxiety regarding infertility (“*psychological baggage of sterility […] is so hard to live with*”). One participant said: "*After recovering from leukemia*, *we are released in the wilds*" “*We are children when we get into the hospital*, *we are adults when we get out*” “*Compared to other children*, *we come from such a different world when we get back to school*”. Psychological support and cooperation among healthcare providers has been showed by Tomioka *et al*. to be a major point of the patient care with HRT [16]. Therefore psychotherapy appears to be instrumental for both infertility acceptance and social reintegration after recovery in this population.

### What we may adjust in our medical practice…

At first HRT initiation, for the endocrinologist to spend time with the adolescent alone seems necessary to explain ovarian failure and treatment outcomes, as along with an organized consultation with a psychotherapist. Besides, since sometimes estrogens patches seemed not generally appreciated leading to poor compliance, personal criteria need to be taken into account when establishing HRT formulation, transdermal *versus* oral.

During long-time follow up, women need repeated information adjusted to their individual situation, according to age, level of understanding, and desire of motherhood. Yearly evaluation of HRT with a trained endocrinologist or gynecologist should be recommended for all patients with discussion dedicated to the treatment: methodical exploration of side effects may improve observance, as well as revaluation of regimen administration with social adjustment. Pointing out positive outcomes of HRT on bone or cardiovascular system could also increase compliance if mentioned. Usual oral HRT may be replaced by contraceptives in case of remaining reproductive function or as per woman’s request. Daily combined pills or transdermal estrogens associated with oral progesterone can easily be prescribed [20]. Etonogestrel implant [21] is interesting in case of chronic diseases and social disorders only in case of proper ovarian function, but should not be prescribed in case of estrogens deficiency. Psychological support remains mandatory in all cases of POI.

This study could lead to the inclusion in the medicine packaging of an accurate notice about the use of HRT for young women mentioning the indication of POI, and not only “*menopause*” which is often taken badly by young women.

This study presents some limits, firstly because of its monocentric character: medical practices remain similar in time especially in hormonology, therefore this study doesn’t give a global overview. Besides, the cohort is heterogeneous: participants had different ages at leukemia diagnosis, various history of puberty evolution before and after HSCT, menarche and pregnancy. Finally, memory of the first HRT prescription and observance was poor because of the interval between puberty induction and study conduction, making this point of interest difficult to explore. The strengths of this study include the single-investigator individual interviews conducted in a short period of time, asking a wide range of questions during the semi-structured interviews to touch on many different issues from pure medical content to psychological aspects including beliefs, fears, and quality of life. This has direct clinical implications in our practice, from prescribing HRT to following up these young women through adolescence and adulthood.

Literature shows that proper adherence requires well-motivated and well-informed women [22]. First, impact of press statements seems essential. HRT received worldwide negative publicity after publication of initial results from Women’s Health Initiative (WHI) trial in 2002 [23] concluding that HRT had more detrimental than beneficial effects [9]. Even if more recent data from randomized trials are consistent with a positive benefit-risk balance for younger women [24, 25] notably regarding both conjugated equine estrogens/medroxyprogesterone acetate and conjugated equine estrogens trials (show a 30% reduction in all-cause mortality when HRT was randomized vs placebo), long-term negative public opinion remains mainly the fear of breast cancer [26, 27]. Physician’s initial advice before starting HRT has been shown to be a major reason for long-term compliance [27, 28], and its continuation appears to be more effective among women who started HRT because of physician recommendations or osteoporosis [29]. Studies also point out that regular verbal counselling is not enough, an educational program seems crucial to avoid premature cessation of treatment [30] with oral and written information adapted to the woman [22]. Not only women but also physicians’ education appears to be decisive to avoid negative attitude regarding HRT [31]. Unacceptable drug-related side effect such as weight gain [22] or irregular bleeding pattern with spotting [32, 33] are some of the main reasons to discontinue HRT that need to be explored further [27]. Therefore, patient-physician communication [26] as well as importance of a concerted choice regarding the galenic formulation both have been mentioned as paramount for a better compliance [34].

In conclusion, teenagers and young women with premature ovarian insufficiency must be taken care of by a multidisciplinary team including pediatricians, hematologists, gynecologists, endocrinologists and psychologists. Hormonal replacement therapy seems closely related to infertility with poor social acceptance; therefore, adherence may be challenging. This qualitative study and review of literature shows that compliance can be improved with initial explanation of valuable outcomes before starting treatment during an individual consultation with the adolescent, educational program and repeated explanation compatible with age and level of comprehension, choice of formulation according to woman personal preference, confidential physician/patient relationship and consequent psychological support during the long-time follow up.

## Supporting information

S1 Appendix Semi-structural interview main items. (DOCX)

## Abbreviations

AMH: Anti-Müllerian Hormone
ESHRE: European Society of Human Reproduction and Embryology
HRT: Hormonal Replacement Therapy
POI: Premature Ovarian Insufficiency
HSCT: Hematopoietic Stem Cell Transplantation
