# Supplementary material for: Misconceptions and beliefs around hormone replacement therapy after childhood hematopoietic stem cell transplantation: A qualitative study among women leukemia survivors
Source: PLoS One. 2023 Apr 11;18(4):e0283940. doi: 10.1371/journal.pone.0283940 (PMC10089326; doi:10.1371/journal.pone.0283940)
Supplement: S1 Appendix — (DOCX) [file pone.0283940.s001.docx]

**APPENDICES**

**Supplementary Material: Semi-structured interview main items**

- **SOCIODEMOGRAPHIC DATA**

**Tell me about you**

How old are you?

Where do you live?

Are you currently in a relationship?

Do you have any children?

- **INITIAL PRESCRIPTION**

**Tell me about the first prescription of hormonal replacement therapy**

How old were you when this treatment was initiated?

For how long have you been taking this medication?

How long (months/years) between the end of leukemia treatment and hormonal treatment initiation?

Why has this treatment been prescribed for you?

What did the doctors explain to you at the beginning of the hormonal treatment?

**What did you understand at first?**

**How did you experience this new treatment initiation?**

- **CURRENTLY**

**How do you feel about this treatment?**

Who is presently prescribing this hormonal treatment for you ?

**How informed do you think you are about this treatment?**

How easy is it to ask questions to your doctor about this treatment?

How easy/difficult is it to discuss this matter with your close relatives?

Have you ever heard about this treatment through the media?

Is it the only treatment you are taking on a daily basis?

Do you also take the contraceptive pill?

- **KNOWLEDGE & BELIEF**

What is your treatment’s name?

**Where do you seek further information if you have questions about the treatment?**

Have you read the patient information leaflet in the box?

Have you looked for information on the Internet?

**What do you personally think about this treatment?**

**What do you think are the benefits of this treatment?**

**How would you feel living without it?**

Do you feel like this treatment is a continuum with the leukemia?

- **OBSERVANCE**

How often do you take your treatment?

**How do you feel after you have forgotten to take it?**

Do you feel any difference when you have taken it and when you have not?

Have you, at least once, totally stopped your treatment without any medical advice? If yes, **please explain why and how you felt.**

- **OBSTACLE & LIMITS ENCOUNTERED**

**What difficulties have you encountered since the treatment started?**

Is it a constraint to take it every day?

**What are the questions you are asking yourself about this treatment?**

Do you think this treatment is safe?

**What fears or apprehensions have you felt about this treatment?**

- **TREATMENT IMPACT ON DAILY LIFE**

Past studies pointed out an impact of the treatment on quality of life and libido. Have you also noticed this in your daily life?

- **CONCLUSION & PERSPECTIVES**

**What would you suggest to improvehormonal treatment observance in young women who experience the same pathology and treatment by chemotherapy?**

Would you like to add something?

Would you like to be kept informed of this study’s results?
